# Removal of sugars in wastewater from food production through heterotrophic growth of *Galdieria sulphuraria*

**DOI:** 10.1002/elsc.202000075

**Published:** 2020-12-21

**Authors:** Philipp Scherhag, Jörg‐Uwe Ackermann

**Affiliations:** ^1^ Department of Chemical Engineering Chair of Bioprocess Engineering and Technical Biochemistry University of Applied Sciences Dresden Dresden Germany

**Keywords:** Galdieria sulphuraria, heterotrophic microalgae, sugar consumption, wastewater

## Abstract

The unicellular extremophilic red alga *Galdieria sulphuraria* is capable of chemoheterotrophy and its growth has been investigated on some defined and undefined substrates. In this study, the removal of sugars in wastewater from fruit‐salad production with *G. sulphuraria* strain SAG 21.92 was analyzed. Growth and sugar consumption were determined under variation of temperature, pH‐value and concentration of a model substrate, containing sucrose, glucose and fructose. In shake flask cultivation maximum specific growth rate and specific substrate consumption rate of 1.53±0.09 day^−1^ and 2.41±0.14 g_Sub_·g_DW_^−1^·day^−1^ were measured at pH 2 and 42°C. A scale‐up of this process was conducted in a 3 L stirred tank reactor (STR). Wastewater from fruit‐salad production was diluted to 15 g·L^−1^ total sugar concentration, supplemented with micronutrients and ammonia and pH was set to 3. Determined growth rate and substrate consumption were 1.21 day^−1^ and 1.88 g_Sub_·g_DW_^−1^·day^−1^, respectively. It was demonstrated, that high sugar concentrations in wastewater streams from food production processes can be significantly reduced with *G. sulphuraria* SAG 21.92. This strain could achieve substrate consumption rates in wastewater, equal to the more common strain 074G, but at higher pH values. Generated biomass can be used for production of phycocyanin, a valuable nutraceutical.

**Abbreviations**
DW: cell dry weight
OD: optical density at 750 nm
STR: stirred tank reactor

## 1 INTRODUCTION

The heterotrophic cultivation of microalgae is gaining in relevance in algae biotechnology [1]. In contrast to photoautotrophic cultivation, biomass is formed in a significantly shorter time due to higher growth rates. Using the same culture media, pH and temperature, a heterotrophic fed‐batch culture of *Neochloris oleoabundans* produced 24 g·L^−1^ cell dry weight, compared with 1 g·L^−1^ under phototrophic conditions in the same time [2]. Additionally no lighting for photosynthesis is required and problems with self‐shading being eliminated [3].

The metabolic capabilities of microalgae make them also suitable for treatment of wastewater [4, 5, 6, 7, 8, 9]. Efficient treatment of wastewater, predominantly measured on removal of N and P, were achieved mostly with chlorophyte like *Chlorella vulgaris, C. reinhardtii, Scenedesmus sp, S. obliquus*, but also with cyanobacteria like *Arthrospira sp*. and *A. platensis* [10, 11, 12, 13]. Besides the crucial removal of N and P, organic compounds in wastewater can be used for transformation into valuable products with microalgae [8, 14, 15, 16]. For example, different species of *Chlorella, Scenedesmus* and *Botryococcus* showed high accumulation of lipids in municipal, agricultural and industrial wastewater, which can be used for biofuel production [11, 17, 18, 19]. Among metabolites like lipids, proteins and polysaccharides, pigments are usually formed in the course of photosynthesis [1]. Most algae lose their pigmentation in the absence of light during heterotrophic growth, but certain species maintain their photosynthetic apparatus even in darkness [8, 20]. Pigment‐concentration of cells grown in the dark are usually lower than under phototrophic conditions, but due to fast heterotrophic growth a great amount of biomass can be obtained during a short time [21]. Volumetric yield of product extracted from harvested heterotrophic biomass is higher, compared to phototrophic cultivation in the same time [22].

However, degradation capacities of wastewater plants are not only limited to N and P but also organic substances. High nutrient loads can lead to increased accumulation in groundwater [7, 16]. Wastewaters from industrial food production processes usually contain high amounts of sugar [23, 24]. Often, they exceed the regulatory discharge levels for dissolved organic carbon and have to be treated further. These high levels of organic substrate are an ideal resource for the production of feedstock or high value products [25]. Utilization of wastewater streams from food production would not only relieve wastewater treatment plants, but is also a step towards a circular economy [26, 27].

*Galdieria sulphuraria* is an extremophilic red alga that is known for their capability of growing on a wide variety of organic carbon sources [28, 29]. Biomass concentrations of 80–120 g·L^−1^ cell dry weight could be obtained with glucose in fed‐batch and continuous‐flow cultivation, which demonstrates that this algae is well suited for high cell density cultivations [30, 31, 32]. This unique metabolism combined with selective growth conditions of pH 1–4 and temperatures up to 56°C makes *G. sulphuraria* a promising organism for the utilization of waste streams [33]. *G. sulphuraria* was already successfully grown on hydrolysates of restaurant and bakery waste [34]. An efficient removal of N and P from urban wastewater under mixotrophic conditions was also achieved with *G. sulphuraria* [35, 36].

In this study, wastewater from a fruit‐salad producer was used as substrate for the heterotrophic growth of *G. sulphuraria* SAG 21.92. Specific growth rate and substrate consumption rate were determined at different pH values, temperatures and sugar concentrations to find optimum process conditions for this strain. The process was than scaled up to a 3 L stirred tank reactor (STR).

**PRACTICAL APPLICATION**

Wastewater from food production contains sugars and other dissolved organic carbon sources. In some cases, regulatory discharge levels for chemical oxygen demand are exceeded because of these substances. A removal can be achieved by heterotrophic conversion into biomass. The extremophilic red alga *Galdieria sulphuraria* is capable of chemoheterotrophic metabolism, besides photoautotrophy. Generated biomass of *G. sulphuraria* can be used for the production of the valuable nutraceutical phycocyanin and the depletion of sugars during growth can be a relief for wastewater treatment plants. In this study we demonstrate, that dissolved sugars in wastewater from fruit‐salad production can be removed by heterotrophic growth of *G. sulphuraria*.

## 2 MATERIALS AND METHODS

### 2.1 Strain and growth media

*Galdieria sulphuraria* strain SAG 21.92 was obtained from Göttingen algae collection (‘Sammlung von Algenkulturen’, Göttingen, Germany). The cells were cultivated in a defined growth medium at pH 2 [29]. Stock cultures were supplemented with 12 g·L^−1^ sucrose, glucose and fructose adding up to a total of 36 g·L^−1^ carbon source and the concentration of ammonium sulfate was increased to 9 g·L^−1^. The resulting C:N ratio was about 7.5:1. The cells were grown in the dark in 250 mL shake flasks containing 50 mL suspension. Temperature and rotational speed were 30°C and 150 rpm (shaking diameter = 25 mm), respectively.

### 2.2 Shake flask cultivation

Growth of *G. sulphuraria* under different conditions was studied in 500 mL shake flasks containing 100 mL suspension. Temperature was varied between 30°C, 42°C, and 50°C, pH‐value between 2.0, 3.0 and 4.0. Sugar concentration was varied in the range between 6 and 60 g·L^−1^, containing equal amounts of the three sugars. All cultivations were carried out under sterile conditions and in the dark at 150 rpm rotational speed (shaking diameter = 25 mm). Evaporation was determined by weight and compensated with sterile water daily.

### 2.3 Stirred tank reactor cultivation

Growth experiments with *G. sulphuraria* were also conducted in a STR containing 3 L suspension. Reactor temperature was set to 42°C, aeration with synthetic air was 3 L·min^−1^ (1 vvm) and the stirrer speed was 150 rpm, respectively. Cultivations were done in defined growth media [29] and in diluted wastewater from fruit salad production. All experiments were conducted under non‐sterile conditions. Evaporation was compensated with water before sampling.

### 2.4 Biomass determination

Optical density (OD) was measured at 750 nm (Specord 40; Analytic Jena AG, Germany) to monitor algal growth. If necessary, samples were diluted below 0.4 with deionized water.

To determine cell dry weight (DW) 900 μL cell suspension were filled into dried, pre‐weighted glass tubes and centrifuged at 13,000 rpm. The supernatant was collected and frozen for determination of ammonia. The cell pellet was dried for 24 h at 100°C. Measurements were always done in quadruples.

### 2.5 Measurement of substrate concentration

Sucrose, glucose and fructose were used as substrate in all experiments and the only sugars present in the wastewater samples. Concentration of these three sugars was measured using high‐pressure liquid chromatography. A Prominence UFLC system (shimadzu corp., Japan) with a PL Hi‐Plex Ca^2+^ 8 μm column was used. Samples of 50 μL were eluted with 0.5 mL·min^−1^ purified water at 80°C, respectively. Sugars were detected with a refraction index detector.

### 2.6 Measurement of ammonia concentration

Ammonia concentration was determined photometrically using an ammonia cuvette test kit (Spectroquant® NH_4_‐N, Or.‐No. 114752, Merck Millipore). Cell‐free supernatant from the biomass determination was treated as instructed and absorbance was measured at 600 nm.

## 3 RESULTS

### 3.1 Characterization of the used wastewater and composition of a synthetic media

All wastewater samples contained sucrose, glucose and fructose, as expected for the procession of raw fruits (see Table 1). Based on these results a model substrate was created containing equal amounts of the three sugars. The term “synthetic media” is used for describing culture media supplemented with the model substrate as carbon source [29]. Total amount of added sugar varied depending on process parameters. Amount of ammonium sulfate was adjusted to C:N‐ratio of 7.5:1. Besides the mentioned sugars, no other substances in relevant concentrations could be detected. The water samples contained no biocidal products or detergents, since cleaning solutions were collected separately. Due to the relatively high sugar concentrations, only the water from the apple‐cleaning bath would be suitable for cultivation without any further dilution.

**TABLE 1:** Sugar content of three different wastewater samples from fruit‐salad production process

Sample	Sucrose [g·L^−1^]	Glucose [g·L^−1^]	Fructose [g·L^−1^]	Total substrate [g·L^−1^]
Sugar‐fond	31.12±1.67^3^	39.02±2.84^3^	32.88±2.65^3^	103.02±7.16^3^
Wastewater from filling process	35.22±1.85^3^	42.67±2.29^3^	36.33±1.85^3^	114.22±5.99^3^
Apple‐cleaning bath	7.33±0.54^3^	5.50±0.63^3^	10.33±1.58^3^	23.17±2.75^3^

Superscript numbers indicate the number of determinations. Error ranges represent standard deviation.

### 3.2 Hydrolysis kinetic due to pH effects

**FIGURE 1:**
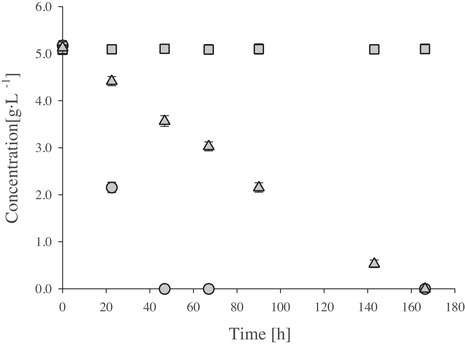
Hydrolysis of sucrose in culture media at 42°C and pH 2 (○), pH 3 (△) and pH 4 (□) Experiment was conducted in 500 mL shake flasks. Error bars represent standard deviation for two experiments

**FIGURE 2:**
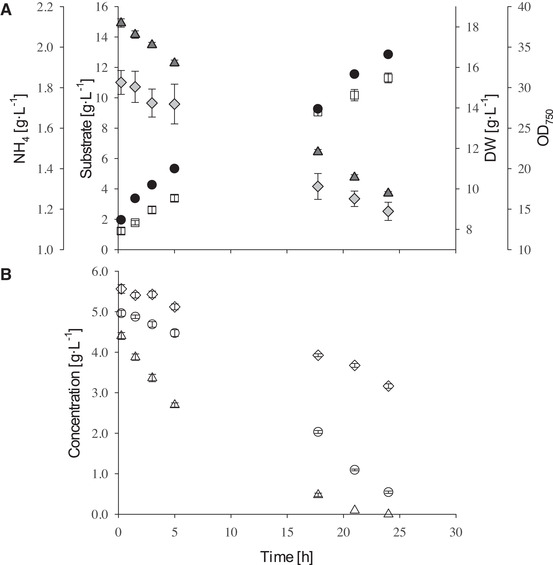
(A) Example for a cultivation of *G. sulphuraria* SAG 21.92 in the STR at 42°C and 15 g·L^−1^ substrate concentration. Measurement of DW (□), OD (●), concentration of substrate (△) and available nitrogen in ammonia (◊) over the course of 24 h. Substrate represents the sum of the three contained sugars sucrose, glucose and fructose. Error bars represent standard deviation. Number of determinations: DW n = 4, OD n = 2, substrate n = 2, ammonia n = 3 (B) Depletion of sucrose (◊), glucose (△) and fructose (○) contained in substrate. Error bars represent standard deviation for two determinations

It is already reported, that microalgae have poor capabilities for disaccharide uptake and lack a transportation system for sucrose in general [5, 37]. *Lepocinclis sp., Euglena mutabilis, Chlorella sacharophila* and *G. sulphuraria* are known to grow on sucrose and it is proposed, that these species produce extracellular hydrolases in order to utilize polysaccharides [29, 38, 39]. However, hydrolysis of sucrose is also catalyzed by protons, which is estimated to mainly contribute to the rate of cleavage. In Figure 1, the hydrolysis rate of sucrose in synthetic media is shown at different pH values. Growth experiments with *G. sulphuraria* showed, that at pH 3 and 4 substrate availability can be limited if glucose and fructose are depleted and sucrose is not completely hydrolyzed (see Figure 2B). Higher cultivation temperatures had a positive effect on sucrose cleavage rate, as expected. At pH 2 and temperatures above 30°C sucrose hydrolysis had no influence on growth, which is in good accordance with previous studies [32].

### 3.3 Optimization of process conditions in shake‐flask scale

The maximum specific growth rate μ_max_ of *G. sulphuraria* SAG 21.92 was 1.53 ± 0.09 day^−1^. It was measured during exponential phase at 42°C, pH 2 and 36 g·L^−1^ substrate concentration (see Table 2). Cultivation temperatures showed the strongest impact on growth of *G. sulphuraria*. Specific growth rate at 42°C was 216% higher than at 30°C and almost 250% higher than at 50°C (at pH 2 and 6 g·L^−1^ substrate concentration). Substrate concentration and pH‐value also affected growth. Compared to pH 2, growth occurred 3% slower at pH 3 and 12% slower at pH 4 on average. At pH 2 the highest growth rates were observed at 36 g·L^−1^ at all three temperatures. Above 36 g·L^−1^ growth rate declined. Similar behavior was observed with *G. sulphuraria* 074G grown on glucose [20]. The highest impact of substrate concentration on growth rate could be observed at 30°C. Compared to 6 g·L^−1^ the specific growth rate at 36 g·L^−1^ (pH 2) was 66% higher (see Table 2).

**TABLE 2:** Specific maximum growth rate μ_max_ and biomass substrate yield Y_X/S_ of *G. sulphuraria* SAG 21.92 in shake flasks at 30°C, 42°C and 50°C

		30°C			42°C			50°C		
Substrate [g·L^−1^]	pH	μ_max_ [day^−1^]	Y_X/S_ [g_DW_·g_Sub_^−1^]	q_S_ [g_Sub_·g_DW_^−1^·day^−1^]	μ_max_ [day^−1^]	Y_X/S_ [g_DW_·g_Sub_^−1^]	q_S_ [g_Sub_·g_DW_^−1^·day^−1^]	μ_max_ [day^−1^]	Y_X/S_ [g_DW_·g_Sub_^−1^]	q_S_ [g_Sub_·g_DW_^−1^·day^−1^]
6	2	0.67±0.11^3^	0.62±0.09^3^	1.14±0.14^3^	1.44±0.04^3^	0.63±0.08^3^	2.28±0.11^3^	0.58±0.05^3^	0.60±0.08^3^	0.96±0.07^3^
6	3	0.69±0.05^3^	0.65±0.12^3^	1.08±0.08^3^	1.39±0.04^3^	0.63±0.06^3^	2.23±0.09^3^	0.57±0.04^3^	0.61±0.07^3^	0.93±0.05^3^
6	4	0.68±0.02^3^	0.61±0.08^3^	1.12±0.08^3^	1.17±0.03^3^	0.63±0.04^3^	1.86±0.05^3^	0.48±0.03^3^	0.62±0.08^3^	0.80±0.07^3^
12	2	0.86±0.07^3^	0.64±0.05^3^	1.35±0.09^3^	1.48±0.09^3^	0.62±0.07^3^	2.39±0.14^3^	0.53±0.05^3^	0.60±0.08^3^	0.88±0.07^3^
12	3	0.84±0.04^3^	0.63±0.05^3^	1.33±0.06^3^	1.39±0.09^3^	0.64±0.06^3^	2.16±0.12^3^	0.54±0.05^3^	0.59±0.07^3^	0.92±0.07^3^
12	4	0.80±0.03^3^	0.60±0.05^3^	1.34±0.08^3^	1.16±0.04^3^	0.65±0.04^3^	1.78±0.06^3^	0.50±0.04^3^	0.58±0.05^3^	0.87±0.04^3^
18	2	0.92±0.10^3^	0.66±0.09^3^	1.34±0.11^3^	n.d.	n.d.	n.d.	n.d.	n.d.	n.d.
30	2	0.92±0.07^3^	0.63±0.05^3^	1.44±0.10^3^	n.d.	n.d.	n.d.	n.d.	n.d.	n.d.
36	2	1.10±0.09^3^	0.65±0.09^3^	1.70±0.11^3^	1.53±0.09^3^	0.64±0.06^3^	2.41±0.14^3^	0.67±0.06^3^	0.52±0.05^3^	1.29±0.10^3^
60	2	0.79±0.06^3^	0.63±0.05^3^	1.25±0.07^3^	1.43	0.61	2.32	0.62±0.08^3^	0.52±0.04^3^	1.18±0.10^3^
60	3	0.74±0.04^3^	0.59±0.03^3^	1.15±0.07^3^	n.d.	n.d.	n.d.	0.55±0.01^2^	0.49±0.04^2^	1.14±0.05^2^
60	4	0.70±0.03^3^	0.59±0.04^3^	1.20±0.07^3^	n.d.	n.d.	n.d.	0.55±0.01^2^	0.43±0.03^2^	1.26±0.04^2^

Resulting specific substrate consumption rate q_S_ was added. Substrate stands for the sum of sucrose, glucose and fructose in equal amounts. Superscript numbers indicate number of experiments. Error ranges represent standard deviation.

Glucose was depleted before fructose. If glucose is present, it is prioritized over fructose by *G. sulphuraria* SAG 21.92 (see Figure 2B).

Variation of biomass substrate yield Y_X/S_ during cultivation experiments was minimal. Higher substrate concentrations seem to lower the biomass yield at 30°C and 50°C, but at 42°C, this trend could not be observed. The lowest yields were measured at 50°C.

Specific substrate consumption rate q_S_ was determined to compare efficiency of sugar depletion. Since the biomass substrate yield nearly remained on the same level, the specific substrate consumption rate showed the same trends as the growth rate. Higher growth rates resulted in higher substrate consumption per g biomass. The maximum specific substrate consumption rate of 2.41±0.14 g_Sub_·g_DW_^−1^·day^−1^ was measured at 42°C, pH 2 with 36 g·L^−1^ substrate.

### 3.4 Scale‐up study in a stirred tank bioreactor

In shake flasks the fastest growth of *G. sulphuraria* was observed at 42°C, 36 g·L^−1^ and pH 2. However, for a whole bioreactor a considerable amount of sulphuric acid is needed to lower the pH‐value of the culture media to the desired level. To minimize the amount of sulphuric acid used for cultivation in the STR, experiments were conducted at pH 3 rather than pH 2. On average a reduction of 3% of the maximum specific growth rate at pH 3 compared to pH 2 was expected, according to data from shake‐flask cultivation. A procession of wastewater in a cycle of 24 h was desirable. In order to achieve complete substrate depletion in the course of 24 h, maximum substrate concentration was limited to 30 g·L^−1^ and starting biomass 8 g·L^−1^ DW was chosen. The specific growth rate and sugar consumption rate were determined at 30 and 15 g·L^−1^ substrate concentration in synthetic media, respectively.

The measured specific growth rate and sugar consumption rate in the STR were not comparable with the data from shake flask cultivation. Both were significantly lower in the STR.

Similar to shake flask cultivation the specific growth rate increased with higher substrate concentration. In the STR the maximum specific growth rate and specific substrate consumption rate of 1.26 ± 0.16 day^−1^ and 2.16 ± 0.19 g_Sub_·g_DW_^−1^·day^−1^, respectively, were measured at 30 g·L^−1^ substrate concentration (see Table 3). Biomass substrate yield and biomass nitrogen yield were higher at 15 g·L^−1^ substrate concentration, compared to 30 g·L^−1^ and ranged between 0.59–0.62 g_DW_·g_Sub_^−1^ and 14.83–15.48 g_DW_·g_NH4_^−1^.

**TABLE 3:** Specific maximum growth rate μ_max_, biomass substrate yield Y_X/S_ and biomass nitrogen yield Y_X/N_. of *G. sulphuraria* SAG 21.92 in the STR at 42°C

Sugar [g·L^−1^]	μ_max_ [day^−1^]	Y_X/S_ [g_DW_·g_Sub_^−1^]	q_S_ [g_Sub_·g_DW_^−1^·day^−1^]	Y_X/N_ [g_DW_·g_NH4_^−1^]
15 (synthetic media)	1.03±0.19^2^	0.62±0.05^2^	1.52±0.11^2^	15.48±0.62^2^
30 (synthetic media)	1.26±0.16^2^	0.59±0.04^2^	2.16±0.19^2^	14.83±0.46^2^
15 (wastewater)	1.21	0.64	1.88	15.19

Resulting specific substrate consumption rate q_S_ was added. Synthetic media contained sucrose, glucose and fructose in equal amounts. Superscript numbers indicate number of experiments. Error ranges represent standard deviation.

Complete depletion of substrate after 24 h could not be achieved with 8 g·L^−1^ starting‐biomass for 15 and 30 g·L^−1^ substrate concentration (see Figure 2A). Besides slower growth of *G. sulphuraria* in the STR, the pH‐value affected substrate availability. As mentioned before, the hydrolysis of sucrose is slower at pH 3 compared to pH 2 (see Figure 1). As a result, after a 24 h cultivation in the STR with 15 g·L^−1^ substrate primarily sucrose was remaining (see Figure 2B).

**FIGURE 3:**
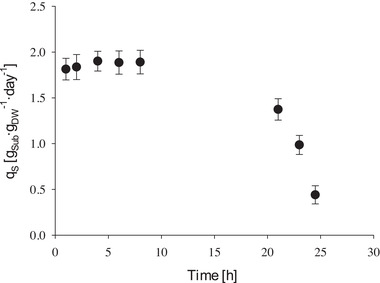
Determined specific substrate consumption rate of *G. sulphuraria* SAG 21.92 in wastewater at pH 3 and 42°C in the STR. Error bars represent standard deviation for two determinations

*G. sulphuraria* SAG 21.92 was successfully grown on wastewater from a fruit‐salad production facility. Wastewater from filling process (see Table 1) was diluted to 15 g·L^−1^ total sugar concentration, micronutrients and ammonia where added accordingly. The specific growth rate in wastewater exceeded growth rate in synthetic media at the same substrate concentration by over 30% and the sugar consumption rate by over 24%. Due to slow hydrolysis rate of sucrose at pH 3, the complete depletion of dissolved sugars could not be achieved. Like in synthetic media, glucose and fructose were almost depleted after 20 h of cultivation and after 24 h, primarily sucrose remained in the wastewater. As a result, specific substrate consumption dropped significantly after 20 h of cultivation in wastewater (see Figure 3). Biomass substrate yield and biomass nitrogen yield were comparable with cultivation using model substrate (see Table 3).

## 4 DISCUSSION

The fastest growth of *G. sulphuraria* SAG 21.92 was observed at 42°C and pH 2, which is in accordance with results of previous works [20, 30, 31, 32, 34]. The determined specific growth rate of 1.53 day^−1^ at 36 g·L^−1^ exceeded all reported growth rates of *G. sulphuraria* strain 074G (see Table 4).

**TABLE 4:** Comparison of specific growth rate and biomass substrate yield of *G. sulphuraria* strain 074G with strain SAG 21.92 (this study) on different substrates

Substrate	Conc. [g·L^−1^]	Temp. [°C]	Reactor system	μ_max_ [day^−1^]	Y_x/s_ [g_DW_·g_Sub_^−1^]	Y_x/n_ [g_DW_·g_NH4_^−1^]	Reference
Glucose	4.5	42	500 mL shake flask	1.10	0.48	11.90	[32]
Fructose	4.5	42	500 mL shake flask	1.08	0.54	11.10	[32]
Glucose	5	42	500 mL shake flask	1.22	0.55	13.7	[34]
Glucose + sucrose	52.5	42	500 mL shake flask	1.44	0.42	11.00	[32]
Glucose + fructose + sucrose	6	42	500 mL shake flask	1.44	0.63	n.d.	This study
Glucose + fructose + sucrose	36	42	500 mL shake flask	1.53	0.64	n.d.	This study
Glucose	10	42	3 L STR	1.26	0.46	n.d.	[31]
Glucose	50	42	3 L STR	1.11	0.55	23.4	[31]
Glucose + fructose + sucrose	15	42	3 L STR	1.03^*^	0.62^*^	15.48^*^	This study
Glucose + fructose + sucrose	30	42	3 L STR	1.26^*^	0.59^*^	14.83^*^	This study

All cultivations were conducted at pH 2, except data marked with ^*^, which were obtained at pH 3.

Variation of different cultivation parameters showed, that the change of the pH‐value had less impact on growth of *G. sulphuraria* SAG 21.92 than the temperature during cultivation. SAG 21.92 seems to adapt better to pH 3, compared to strain 074G. Specific growth rate of SAG 21.92 at 42°C was on average 3% lower at pH 3 than at pH 2. For strain 074G a decline in specific growth rate of about 14% was reported [40]. To reduce the amount of sulphuric acid needed for cultivation the elevation of pH‐value might thus be reasonable. Above 36 g·L^−1^ substrate concentration a slight decrease in growth rate was observed at all three temperatures. Similar behavior was reported for *G. sulphuraria* 074G [20]. Other studies observed lower growth rates at 50 g·L^−1^ compared to 5 g·L^−1^ glucose for strain 074G [30, 31]. In contrast Schmidt and colleagues reported a decrease of the growth rate starting above 166 g·L^−1^ glucose [32]. Determined biomass substrate yield and biomass nitrogen yield also showed good accordance with previous works on *G. sulphuraria* 074G (see Table 4).

The variation of pH value also revealed problems with treatment of wastewaters containing higher concentration of sucrose. Since the hydrolysis rate of sucrose is temperature dependent, a procession of wastewater containing considerable amounts of sucrose is not recommended at 30°C without any further treatment. To overcome problems of sucrose cleavage a storage tank for wastewater should be established. In advance of the cultivation with *G. sulphuraria*, the wastewater is collected and treated with sulphuric acid at 42°C to induce sucrose hydrolysis. Determined hydrolysis rate of sucrose was lower in cell‐free media in shake‐flasks compared to cultivation in the STR. The amount of hydrolyzed sucrose after 24 h cultivation in the STR was on average 79% higher (data not shown) than in the cell‐free synthetic media in shake‐flasks. Besides acceleration of hydrolysis due to acidification of the culture media caused by ammonia uptake, the presence of extracellular hydrolases could be an explanation [40].

Expected growth rates from shake flask cultivation could not be reached in the STR. Since oxygen supply was sufficient (data not shown), deficits could be caused by cells aggregating on the reactor wall due to foaming. Cultivations with wastewater exceeded growth rates in synthetic media with 15 g·L^−1^ total sugar concentration in the STR. The synthetic media contained equal amounts of the three sugars, whereas the wastewater from filling process contained more glucose than sucrose (see Table 1). Sucrose needed to be hydrolyzed before consumption. At 15 g·L^−1^ substrate concentration this cleavage reaction was growth limiting. Because of the lower sucrose concentration in the wastewater compared to the synthetic media, *G. sulphuraria* SAG 21.92 could grow faster in the wastewater.

Determined specific growth rate of 1.21 day^−1^ of strain SAG 21.92 in wastewater supplemented with micronutrients and ammonia was in good accordance with previous studies with strain 074G in hydrolyzed restaurant waste (also supplemented with micronutrients and ammonia), where a specific growth rate of 1.22 ± 0.01 day^−1^ was obtained [34]. Resulting sugar consumption rates were almost identical. Utilization of hydrolyzed potato starch with *G. sulphuraria* was also shown recently, where a specific growth rate of 0.82 day^−1^ was attained [41]. The mentioned growth rates were measured at pH 2 in contrast to this study, where comparable growth rates could be achieved at pH 3 with strain SAG 21.92. In process scale, this could save a significant amount of sulphuric acid. Recovery of sulphuric acid from used wastewater is possible, but optimization has to be addressed in further investigations. To date, no other studies have investigated biomass production of *G. sulphuraria* with waste streams containing high loads of organic carbon.

However, other microalgae like *Chlorella zofingiensis* or *C. protothecoides* could already be cultivated in waste molasses for pigment and biofuel production [42, 43]. Also dairy effluent has proven to be a suitable carbon source for *Chlorococcum sp* [44]. The authors propose that *G. sulphuraria* could also harness the carbon sources in the mentioned waste streams. *G. sulphuraria* is also able to produce the pigment phycocyanin. A phycocyanin production process based on organic carbon in wastewaters from food production process with *G. sulphuraria* will be investigated in future studies.

## CONFLICT OF INTEREST

The authors have declared no conflict of interest.

## Nomenclature

**Table:** 

μ_max_	[day^−1^]	Maximum specific growth rate during exponential phase
Y_X/S_	[g_DW_·g_Sub_^−1^]	Biomass substrate yield
Y_X/N_	[g_DW_·g_NH4_^−1^]	Biomass nitrogen yield
q_S_	[g_Sub_·g_DW_^−1^·day^−1^]	Specific substrate consumption rate

## Data Availability

The data that support the findings of this study are available from the corresponding author upon reasonable request.
